# Carrier Trap and Their Effects on the Surface and Core of AlGaN/GaN Nanowire Wrap-Gate Transistor

**DOI:** 10.3390/nano13142132

**Published:** 2023-07-22

**Authors:** Siva Pratap Reddy Mallem, Peddathimula Puneetha, Dong-Yeon Lee, Yoonkap Kim, Han-Jung Kim, Ki-Sik Im, Sung-Jin An

**Affiliations:** 1 Advanced Material Research Center, Kumoh National Institute of Technology, Gumi 39177, Republic of Korea; drmspreddy@kumoh.ac.kr; 2 Department of Robotics and Intelligent Machine Engineering, College of Mechanical and IT Engineering, Yeungnam University, Gyeongsan 38541, Republic of Korea; puneethaphd@gmail.com (P.P.); dylee@ynu.ac.kr (D.-Y.L.); 3 Nano Electronic Materials and Components Research Center, Gumi Electronics and Information Technology Research Institute (GERI), Gumi 39171, Republic of Korea; yoonkap@geri.re.kr (Y.K.); hjkim0321@geri.re.kr (H.-J.K.); 4 Department of Green Semiconductor System, Korea Polytechnics, Daegu Campus, Daegu 41765, Republic of Korea; 5 Department of Materials Science and Engineering, Kumoh National Institute of Technology, Gumi 39177, Republic of Korea

**Keywords:** carrier trap, 1/*f*-noise, nanowire, AlGaN/GaN

## Abstract

We used capacitance–voltage (*C*–*V*), conductance–voltage (*G*–*V*), and noise measurements to examine the carrier trap mechanisms at the surface/core of an AlGaN/GaN nanowire wrap-gate transistor (WGT). When the frequency is increased, the predicted surface trap density promptly drops, with values ranging from 9.1 × 10^13^ eV^−1^∙cm^−2^ at 1 kHz to 1.2 × 10^11^ eV^−1^∙cm^−2^ at 1 MHz. The power spectral density exhibits 1/*f*-noise behavior in the barrier accumulation area and rises with gate bias, according to the 1/*f*-noise features. At lower frequencies, the device exhibits 1/*f*-noise behavior, while beyond 1 kHz, it exhibits 1/*f*^2^-noise behavior. Additionally, when the fabricated device governs in the deep-subthreshold regime, the cutoff frequency for the 1/*f*^2^-noise features moves to the subordinated frequency (~10^2^ Hz) side.

## 1. Introduction

Nanoscale circuit components such as solid-state nanowires have significantly advanced because of their prospective use in future-generation, high-performance electronic and optoelectronic appliances [1,2,3,4,5,6,7]. Nanowire-based technologies for developing wrap-gate transistors (WGTs) have been demonstrated effectively compared to conventional field-effect transistors (FETs) [8,9,10,11]. Investigations have also been conducted on nanowire WGTs produced through a top-down fabrication method using a sacrificial layer contrary to the bottom-up method. This top-down approach has many benefits, such as the ability to (i) generate many lateral nanowires with fine alignment, (ii) reduce device dimensions, and (iii) manufacture a wide-ranging comprehensive structure with high yield. 

Normally, GaN-based nanowire WGTs are often appealing for high-performance functions like high-power, high-speed, high-frequency, and high-temperature devices. The difficulty of etching GaN using the wet/dry etching method makes AlGaN/GaN-based nanowire WGT interesting. In the present study, we fabricate and characterize one-dimensional AlGaN/GaN nanowire WGT on a GaN-on-insulator (GaNOI) wafer via the top-down method. AlGaN/GaN-based nanowire WGTs are attractive devices for high-power/high-frequency applications due to the inherent material characteristics related to III-V semiconductors, such as a wide band gap, large breakdown voltage, and high saturation velocity. 

The electrical characteristics of these nanowire devices are characteristically limited by the carrier defects/traps originating at the surface and core of the nanowire. In fact, carrier traps or deep levels originate near the energy of the mid-gap and serve as generation-recombination (*G*–*R*) centers that can influence the carrier transport [12,13,14,15]. Using optimum growth conditions and appropriate substrates, the creation of these traps, as well as the electrically active defects, must be minimized or regulated [16]. Post-growth processing processes like thermal annealing can be used to further reduce the density/concentration of the traps associated with the deep levels as well as point defects [17]. Therefore, it is essential to comprehend trap behavior in order to manage the electrical performance of nanowire devices. An earlier study [18] reported trap and 1/*f*-noise effects at the surface and core of GaN nanowire WGTs. In the present study, the frequency-dependent capacitance–voltage (*C*–*V*) and conductance–voltage (*G*–*V*) measurements, as well as the associated 1/*f*-noise characteristics, are crucial for comprehending the impacts of trapping at the surface and/or in the core of the AlGaN/GaN nanowire channels on the performance of the device. 

The primary goal of this research is to examine the carrier trap feature at the surface and core of the nanowire using capacitance–voltage (*C*–*V*) and conductance–voltage (*G*–*V*) characteristics observed in the frequency range from 1 kHz to 1 MHz. The estimation of surface trap density follows as a secondary goal. Finally, the one-dimensional AlGaN/GaN nanowire WGTs 1/*f*-noise properties are assessed. 

## 2. Materials and Methods

For the GaN nanowire WGT design, we used a 4-inch diameter GaNOI substrate made by SOITEC Corporate utilizing the Smart CutTM method and double-wafer transfer technology. A sapphire (0.65 mm-thick) substrate was covered with a GaN (150 nm-thick) layer and buried oxide (SiO_2_, 800 nm-thick) to create a 4-inch diameter GaNOI substrate. The {11–20} crystal orientation was initially formed on the GaNOI substrate using a sophisticated electron beam (e-beam) lithography technique with PMMA resist. GaN film (150 nm) was selectively etched with an etching width of 0.32 μm using an inductively coupled plasma process for approximately 70 s at an etch rate of 2 nm/s, and then the device-patterned wafer was etched with TMAH solution for 10 min at 80 °C. This etchant solution only etches in the lateral crystal direction, not the vertical *c*-plane (0001) crystal direction. Here, TMAH etching caused the pattern GaN nanowire’s width to gradually decrease while maintaining its trapezoidal shape. The substrate was also immersed in a buffer-oxide etchant (BOE) solution to successfully remove the buried oxide behind the GaN nanowires. Additionally, using the metal–organic chemical vapor deposition (MOCVD) method selectively grew undoped 50 nm-thick GaN/20 nm-thick AlGaN layers on the GaN pattern. Here, a self-limiting regrowth process in the {1–101} orientation produced an *r*-plane on the patterned GaN layer. Surprisingly, the surface of the r-plane is composed of nitrogen (N) atoms that easily formed N-H bonds by interacting with hydrogen (H) atoms in the MOCVD chamber. Development was constrained by this interaction, which also strengthened stability in the plane direction. Only at the source and drain regions, as well as on the top side of the GaN nanowire, could AlGaN/GaN films be produced so easily again. 

Gate electrode (10 nm-thick of TiN) and high-k gate oxide (20 nm-thick of Al_2_O_3_) were gradually coated for the manufacture of WGT devices using the plasma-enhanced atomic layer deposition (PE-ALD) method. A four-layer metal stack (Ti/Al//Ni/Au) was subsequently deposited as source/drain areas using the e-beam method, followed by quick thermal annealing in an N_2_ atmosphere at 850 °C for 30 s. In order to act as an external contact for electrical properties, a gate metal layer coated with Ni/Au was applied last. The carrier concentration and mobility of the regrown AlGaN film were measured using a Hall-effect measurement instrument (HL5500PC, Nanometrics, Milpitas, CA, USA), measuring 9.7 × 10^12^ cm^−2^ and 1640 cm^2^/Vs, respectively. The device architecture and element identifications were obtained using a field-emission transmission electron microscope (FE-TEM, JEM-2100F, JEOL, Tokyo, Japan) with scanning transmission electron microscopy (STEM) arrangement. A semiconductor source system (B1500, Agilent, Santa Clara, CA, USA) was used to measure the *C*–*V* and *G*–*V* properties of one-dimensional AlGaN/GaN nanowire WGTs. A low-frequency noise tracking system (Synergie Concept/Instrumentation & Electronic, Meylan, France) was used to measure the 1/*f*-noise. 

## 3. Results

**Figure 1:**
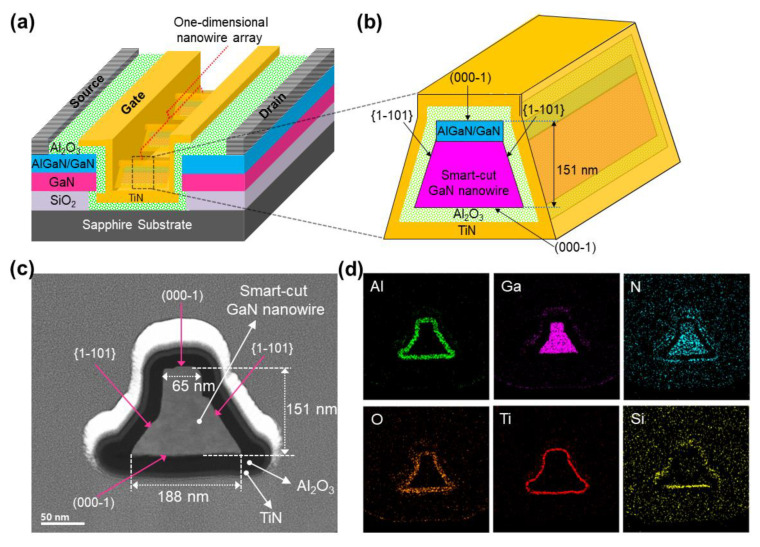
(**a**) Graphical device design of the fabricated AlGaN/GaN nanowire WGT. (**b**) Cross-section image of a trapezoidal-shaped AlGaN/GaN nanowire. (**c**) High-resolution FE-TEM image of the AlGaN/GaN nanowire WG structure. (**d**) Elemental spatial mapping of Al (green), Ga (pink), N (cyan), O (orange), Ti (red), and Si (yellow) in the device design scanned using STEM-EDX.

Figure 1a (on the left side) illustrates the schematic architecture of the investigated AlGaN/GaN nanowire WGT device. It has a 2-μm gate length consisting of 64 trapezoidal shaped one-dimensional nanowires, each having two similar {1–101} crystal facets (Figure 1b). On the left side of Figure 1c, an FE-TEM image clearly shows a trapezoidal-shaped AlGaN/GaN nanowire core surrounded by gate–oxide (Al_2_O_3_) and gate–metal (TiN). Figure 1d shows the spatial elemental mapping distributions of Al, Ga, N, O, Ti, and Si in the fabricated device architecture, which are examined using STEM consists with energy-dispersive X-ray (EDX) arrangement to verify the self-limited growth of the AlGaN/GaN nanowire WG architecture. 

**Figure 2:**
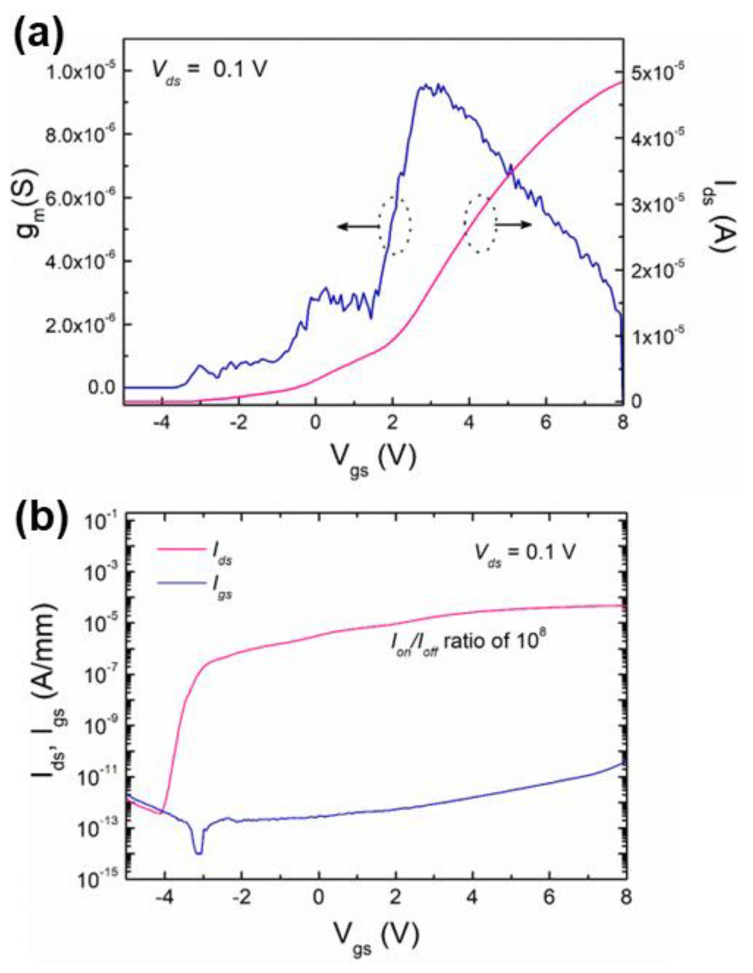
(**a**) Transconductance (*g*_m_) and linear scales of the drain current (*I*_ds_) as a function of gate-source voltage (*V*_gs_). (**b**) Logarithmic scales of drain current (*I*_ds_) as well as the gate current of (*I*_gs_) according to the *V*_gs_ of the measured AlGaN/GaN nanowire WGT at *V*_ds_ = 0.1 V.

Figure 2a shows that the transistor exhibits normal operation with a threshold voltage (*V*_th_) of ∼−1.5V. The obtained threshold voltage may be due to the full depletion of the lateral trapezoidal-shaped cross-section of the narrow channel. The maximum drain current (*I*_d,max_) of nearly ∼0.05 mA and maximum transconductance (*g*_m_) peak of nearly ∼0.01 mS at *V*_gs_ = 8 V and *V*_ds_ = 0.1 V are exhibited. Furthermore, the semi-logarithmic transfer characteristics are shown in Figure 2b. The device leakage current is as low as ∼10^−11^ A∙mm^−1^. The device exhibits a high *I*_on_/*I*_off_ ratio of 10^8^.

**Figure 3:**
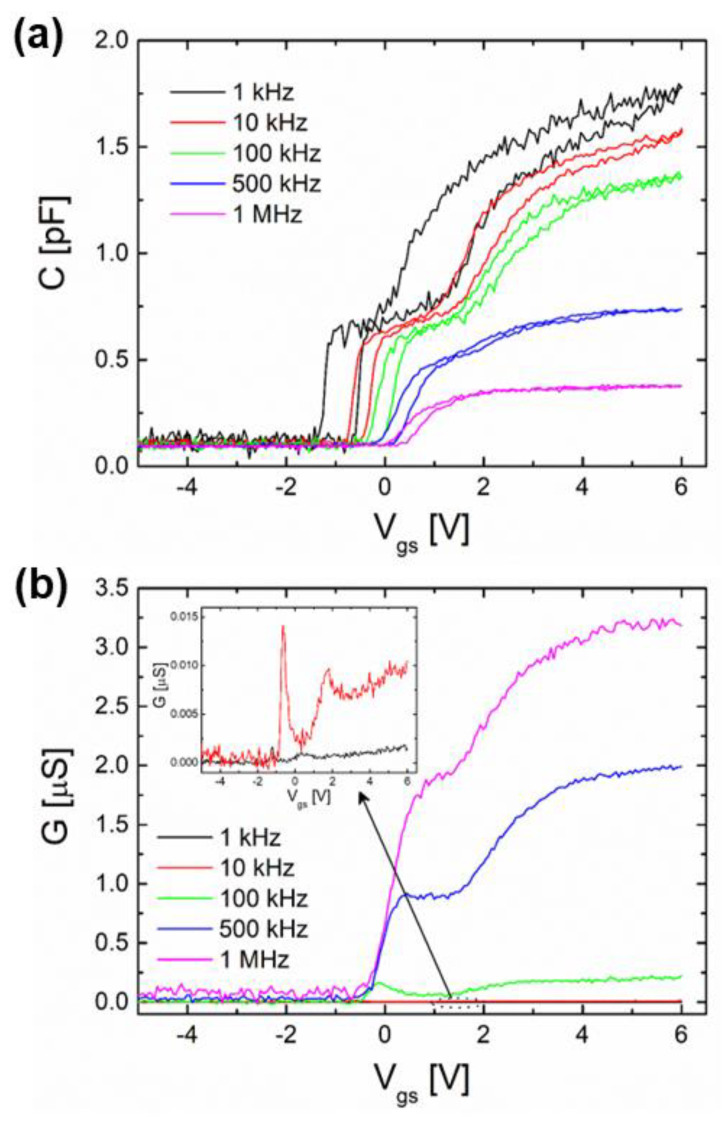
(**a**) *C* versus *V*_gs_ and (**b**) *G* versus *V*_gs_ as functions of frequency of the AlGaN/GaN nanowire WGT.

Figure 3a shows the *C*–*V* measurements of AlGaN/GaN nanowire WGT measured from low frequency (1 kHz) to high frequency (1 MHz). The measured *C*–*V* curve exhibits four different bias regions, such as barrier accumulation, 2DEG accumulation, surface depletion, and total depletion of the whole nanowire channel. Generally, the surface inversion is missing because of the significantly long-lasting minority carrier generation that occurs in GaN-based materials [18,19]. It is observed that the barrier accumulation happens at an immense positive bias shift of the flat-band voltage (*V*_FB_), with drastic frequency distribution as frequency rises. In addition, the change in flat-band voltage (Δ*V*_FB_) shift in hysteresis curves drops with rising frequency, and values are shown in Table 1. The observed strong frequency dispersion on *C*–*V* curves at the accumulation is mainly due to the trapping of electrons at border traps in the Al_2_O_3_ insulator. Such phenomena, i.e., overestimation of the oxide capacitance at the accumulation due to a decrease in frequency, were previously observed in many oxide/III-V semiconductor devices [20]. In addition, this extreme frequency dispersion is caused by a large number of surface-related traps with different lifetimes. The associated interface trap capacitance (*C*_it_) is substantial, and the surface traps respond well to low/intermediate-level alternating-current (AC) wave frequencies, but not well to high frequencies [18,21]. The dispersion may also be caused by the deep traps that were additionally injected into the GaN film at the time of the wafer bonding method. High frequency causes a serious scattering in the barrier accumulation layer and a high positive bias shift of *V*_FB_ with enlarging frequency because the trapped carriers are unable to react to the AC wave and are, therefore, unable to allow the current motion. 

Figure 3b presents the *G*–*V* characteristics for the measured nanowire device. The *G*–*V* curve decreases to negative bias, but rises with enhancing frequency, oppositely to the *C*–*V* characteristics. Additionally, it is noted that the *G*–*V* peak exists at a lower frequency range (~1 to 100 kHz) but vanishes at a higher frequency range (~500 to 1000 kHz). The surface traps with variable time responses, as mentioned in *C*–*V* characteristics, are the exact same source from which the conductance behavior derives. This states that at low frequencies, the surface traps perhaps obey the frequency and then contribute obviously to the fabricated device conductance (the period of frequency is larger than the lifetime of trapped carriers) [18,22,23]. The trapped carriers almost cannot obey the AC wave at adequately high frequencies (>500 kHz), opposite to lower frequency ranges, and do not devote to the consistent conductance. Particularly, in contrast to low frequencies, the time constant at high frequencies is substantially bigger than the period, making it impossible for the charges at traps to obey the AC wave. 

**Figure 4:**
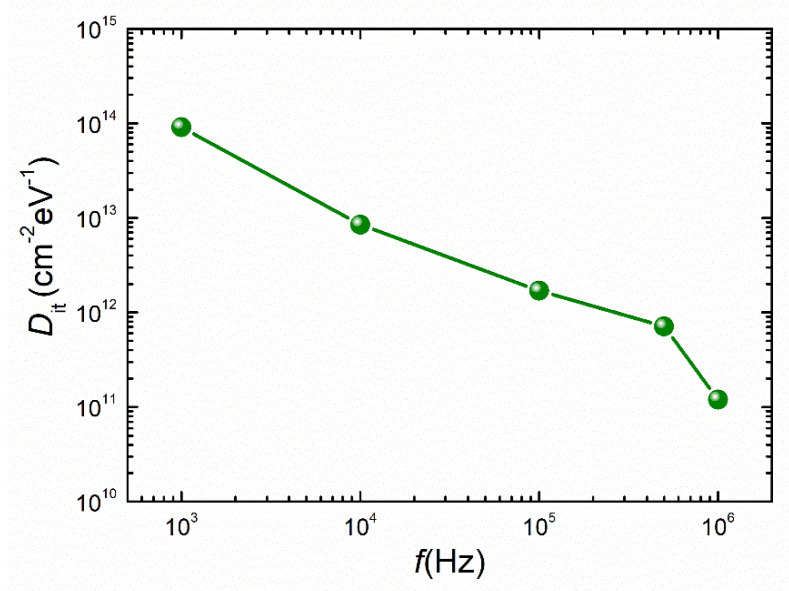
The effective *D_it_* versus frequency for the AlGaN/GaN nanowire WGT.

The interface state density (*D*_it_) is calculated using the Hill–Coleman phenomena [24], such that

(1) $$D_{it}=\frac{2}{qA}\left[\frac{{\left(G/\omega \right)}_{max}}{{\left(C_{OX}{\left(G/\omega \right)}_{max}\right)}^{2}+{\left({1-C/C}_{OX}\right)}^{2}}\right]$$

where *q* is the electron charge, and *A* is the area of contact. *C_OX_* is the oxide capacitance, which can be evaluated from capacitance and conductance characteristics in strong accumulation regions at high-frequency regimes. *D_it_* is evaluated using Equation (1) and values of 9.1 × 10^13^ (at 1 kHz), 8.5 × 10^12^ (at 10 kHz), 1.7 × 10^12^ (at 100 kHz), 7.1 × 10^11^ (at 500 kHz), and 1.2 × 10^11^ eV^−1^∙cm^−2^ (at 1 MHz) were obtained, respectively. In a similar range of values found from oxide-based III–V semiconductor interfaces [18,19,20], the obtained *D_it_* values drop with increasing frequency, as shown in Figure 4. More precisely, if the frequency decreases, more traps are located at a deeper response. The strong dispersion influences on the *C*–*V* and *G*–*V* properties are caused by the high value of *D_it_*. 

**Figure 5:**
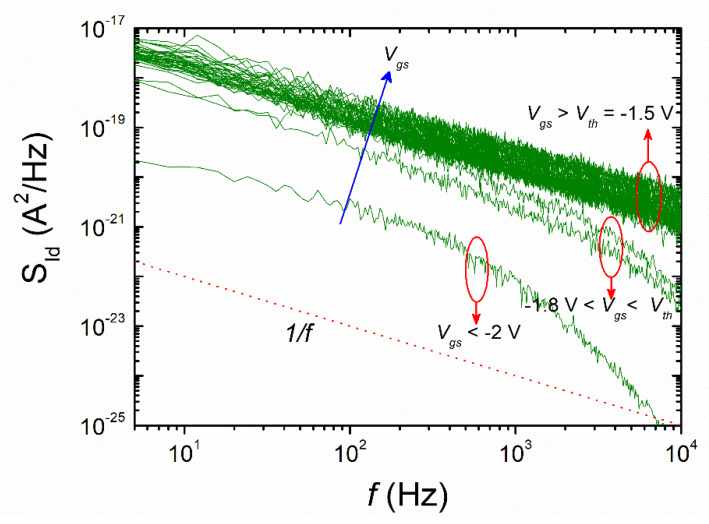
Typical power spectral distribution (PSD) of low-frequency noise for the measured AlGaN/GaN nanowire WGT (at *V*_ds_ = 0.1 V).

To further clearly investigate the trapping effect through the noise measurements, low-frequency noise tests are carried out to more thoroughly examine the trapping effect in the one-dimensional AlGaN/GaN nanowire WGT. It is widely known that 1/*f*-noise plots can provide precise information on the nature of the carrier conduction at the surface/bulk of the materials when combined with other electrical measurements. To investigate impurities and defects with deep-level traps in bulk materials and to assess the suitability (i.e., for microwave communication systems), standard quality, and reliability of semiconductor-based devices, low-frequency noise characteristics are employed [25,26]. When the gate bias is changed from the deep subthreshold to a strong accumulation regime, the power spectral density (PSD, S_Id_) of the current/voltage dispersions is measured at *V*_ds_ = 0.1 V and a frequency range of 4–10^4^ Hz. As seen in Figure 5, the PSD elevates to the gate voltage. The noise plots distinctly display a 1/*f*-plot shape in the strong accumulation region (*V*_gs_ > *V*_th_ = −1.5 V), indicating that the noise is primarily caused by electron movements between the surface states with shallow levels and the accumulated surface channel layer, as mentioned before in the capacitance and conductance measurements. In the depletion region (−1.8 V < *V*_gs_ < *V*_th_), the device exhibits 1/*f*-noise behavior, while beyond 1 kHz, it exhibits 1/*f*^2^-noise characteristics (i.e., Lorentzian-like (1/*f*^γ^, γ = 2)). The *G*–*R* noise that results from carrier transfer processes via trap/detrap between the 2DEG channel and traps in the GaN film with approximately shorter time constants is believed to be the cause of these 1/*f*^2^ characteristics [27,28]. This indicates that the carriers know how trapping and detrapping alike are at the interface and in the surface depletion layer of the channel. At *V*_gs_ < −2 V (i.e., deep-subthreshold regime), the cutoff frequency for the 1/*f*^2^-noise curve moves to a lower frequency (∼10^2^ Hz) edge. This behavior is due to the *G*–*R*-based carrier transfer mechanism with comparably longer lifetime constants. 

## 4. Conclusions

In summary, with the help of frequency-dependent capacitance–voltage (*C*–*V*) and conductance–voltage (*G*–*V*) measurements, the trapping effects and low-frequency noise measurements of trapezoidal-shaped lateral one-dimensional AlGaN/GaN nanowire WGTs fabricated using the top-down method are examined. The device displays electrical characteristics such as a high *I*_on_/*I*_off_ ratio of around 10^8^. It has been shown that the device’s capacitance, conductance, and noise properties are significantly influenced by the high trap density present both at the nanowire channel’s surface and in its core. According to the 1/*f*-noise plots, the power spectral density displays 1/*f*-noise behavior in the accumulation regime and rises with gate bias. The device displays 1/*f*-noise behavior at low-frequency range and 1/*f*^2^-noise behavior at 1 kHz. The cutoff frequency for the 1/*f*^2^-noise plots also shifts to the lower frequency (10^2^ Hz) side when the nanodevice rules in the deep-subthreshold regime. Our findings are expected to be exploited for the evolution of high-performance one-dimensional AlGaN/GaN nanowire WGTs.

## Figures and Tables

**Table 1:** Calculated experimental parameters obtained from capacitance and frequency measurements.

Frequency, *f* (kHz)	*V*_FB_ (V)	Δ*V*_FB_ (V)	*D_it_* (eV^−1^∙cm^−2^)
1	−1.21	0.72	9.1 × 10^13^
10	−0.60	0.33	8.5 × 10^12^
100	−0.16	0.28	1.7 × 10^12^
500	0.17	0.24	7.1 × 10^11^
1000	0.28	0.21	1.2 × 10^11^

## Data Availability

The data are available on reasonable request from the corresponding author.
